# A Presence-Only Model of Suitable Roosting Habitat for the Endangered Indiana Bat in the Southern Appalachians

**DOI:** 10.1371/journal.pone.0154464

**Published:** 2016-04-26

**Authors:** Kristina R. Hammond, Joy M. O’Keefe, Stephen P. Aldrich, Susan C. Loeb

**Affiliations:** 1 Center for Bat Research, Outreach, and Conservation, Indiana State University, Terre Haute, Indiana, United States of America; 2 Department of Earth and Environmental Systems, Indiana State University, Terre Haute, Indiana, United States of America; 3 United States Department of Agriculture, Forest Service, Southern Research Station, Clemson, South Carolina, United States of America; Università degli Studi di Napoli Federico II, ITALY

## Abstract

We know little about how forest bats, which are cryptic and mobile, use roosts on a landscape scale. For widely distributed species like the endangered Indiana bat *Myotis sodalis*, identifying landscape-scale roost habitat associations will be important for managing the species in different regions where it occurs. For example, in the southern Appalachian Mountains, USA, *M*. *sodalis* roosts are scattered across a heavily forested landscape, which makes protecting individual roosts impractical during large-scale management activities. We created a predictive spatial model of summer roosting habitat to identify important predictors using the presence-only modeling program MaxEnt and an information theoretic approach for model comparison. Two of 26 candidate models together accounted for >0.93 of AICc weights. Elevation and forest type were top predictors of presence; aspect north/south and distance-to-ridge were also important. The final average best model indicated that 5% of the study area was suitable habitat and 0.5% was optimal. This model matched our field observations that, in the southern Appalachian Mountains, optimal roosting habitat for *M*. *sodalis* is near the ridge top in south-facing mixed pine-hardwood forests at elevations from 260–575 m. Our findings, coupled with data from other studies, suggest *M*. *sodalis* is flexible in roost habitat selection across different ecoregions with varying topography and land use patterns. We caution that, while mature pine-hardwood forests are important now, specific areas of suitable and optimal habitat will change over time. Combining the information theoretic approach with presence-only models makes it possible to develop landscape-scale habitat suitability maps for forest bats.

## Introduction

Forests are important to many bat species in North America as both roosting and foraging habitat [1]. However, the high mobility of bats creates a problem for land managers seeking to identify critical habitat within forests [1–2]. Because locating bat roosts is costly and time consuming, it can be difficult to plan timber harvests and prescribed fires that protect or create habitat for federally endangered species, such as the Indiana bat *Myotis sodalis* [3]. In large remote areas, such as national forests, there is a critical need for predictive tools that map the distribution of important habitats for bats. Although many bat species have been tracked to individual roost sites and important characteristics at the tree and stand scales have been identified [1], [4], there are few data on characteristics of suitable roosting sites at the landscape scale (e.g., [5]). *Myotis sodalis* roost primarily in snags, but protecting all potential roosts is not a practical management strategy [3], [6–7]. Rather, it is critical to identify important environmental variables that can be used to predict roost locations. Our objective was to develop a landscape-scale model to predict the location of potential *M*. *sodalis* summer roosting habitat within our study area in the southern Appalachian Mountains.

Since its listing in 1967, agencies have tried to conserve and manage habitat thought to be critical to *M*. *sodalis* [3], which is widely distributed across much of eastern North America. Before 1995, most National Forest plans focused on protecting hibernacula and preserving large diameter mature hardwood trees in riparian areas [3], the latter having been found to be critical *M*. *sodalis* summer habitat in the Midwest [6]. However, in 1994, a *M*. *sodalis* was tracked to upland habitat in Kentucky [3], leading to more intensive surveys in the southeastern U.S. (e.g. [8]). We now know that in the southern Appalachian Mountains, Indiana bats maternity colonies typically roost in dead pine trees near a ridge top [9], [this study], which is a very different from the standard definition of critical habitat based on studies in the Midwest [5–6]. It is clear that *M*. *sodalis* are not restricted to riparian zones for roosting and that females are reproducing farther south than previously known [3], [10].

To create a landscape-scale predictive model of the distribution of *M*. *sodalis* summer roosting habitat, it is important to consider forest patch characteristics (e.g., patch size, heterogeneity, canopy closure, and snag density), topographic variables (e.g., elevation, aspect, and slope), and proximity to foraging areas or water [11]. Kalcounis-Rueppell et al. [4] concluded that proximity to canopy openings, proximity to other snags, and proximity to water were important factors in roost site selection for forest bats in North America; however, across our study area in the southern Appalachian Mountains data are insufficient for mapping canopy gaps and snag availability. Instead, we used data on forest composition, topographic features, and proximity to foraging areas or water as predictors of the distribution of roost habitat across the forested landscape of our study area. Most of the area is heavily forested, with a mix of hardwood and pine trees [12]. The topography is mountainous and rugged, and vegetative communities vary by elevation, aspect, and degree of slope [12]. We considered topographic factors in our models because reproductive female bats often roost at lower elevations and in areas with high solar exposure to help ensure stable and warm roost conditions for their young [11, 13]. Distance to foraging areas or water may also be important for reproductive females seeking to minimize commuting costs to prey-rich areas or to water sources.

To develop a habitat distribution model for *M*. *sodalis*, we employed MaxEnt, which is a presence-only modeling approach that estimates distribution based on known locations and background sampling of the environment [14–15]. MaxEnt is a hypercomplex regression technique equivalent to Poisson regression that uses random background absence points for model calibration [16]. MaxEnt tolerates low sample sizes (i.e., <100), although larger sample sizes are desirable when this equates to better coverage of the study area [17]. Outputs can be transferred to geographic information systems (GIS), allowing for interpretation of the output in a simple visual format [18]. MaxEnt has been used for predicting large-scale distributions of some bat species, including *Barbastella barbastellus* distribution in Portugal [19] and Italy [20], and current and potential distribution of *Myotis simus* in South America [21]; both species are in the family Vespertilionidae, as is *M*. *sodalis*. Loeb and Winters [22] used MaxEnt to assess the influence of climate change on the distribution of *M*. *sodalis* across the entire species’ range. MaxEnt can also be used to model species distributions at more local scales. Baldwin and Bender [23], for example, input topographic and other environmental variables into MaxEnt to model the distribution of denning habitat for *Ursus americanus* in Rocky Mountain National Park, Colorado, USA. Bellamy and Altringham [24] entered roost records into MaxEnt to create habitat suitability models for eight bat species in a ~2,300 km^2^ area in England. Likewise, we used variables related to both biotic and abiotic factors [25] in MaxEnt models to predict the distribution of suitable roosting habitat for *M*. *sodalis* in our southern Appalachian study area (~2,800 km^2^).

MaxEnt has a few weaknesses that are important to consider. First, the final product is not easily transferred to non-sampled areas or areas where environmental conditions differ significantly [15], [18]. However, models created by Maxent can identify important environmental variables and information obtained during the modeling process may inform models developed other areas. It can also be difficult to find an appropriate method to evaluate competing models. To evaluate and compare models, we used an information theoretic (IT) approach to reduce the likelihood of over parameterization and to avoid problems associated with using area under the curve (AUC) scores that are output by MaxEnt [26]. AUC scores may be misleading for several reasons [26], and do not account for number of parameters and over fitting of data [27–28]. Using an IT approach allowed us to compare a suite of plausible yet simple *a priori* hypotheses [29]; with the exception of the global model, each model we tested was built from only a subset of the 10 environmental variables we measured to predict the distribution of *M*. *sodalis* habitat in the southern Appalachians.

Our aim was to create a model of *M*. *sodalis* summer roosting habitat distribution within a portion of the southern Appalachian Mountains and to identify environmental predictors that might be important in other landscapes. We sought to generate a GIS layer that could be used by land managers to understand the probability of presence of Indiana bat roosting habitat across a 5 county area in the southern Appalachians. Based on 5 years of field observations, we expected that important areas for summer roosting habitat would be concentrated on south-facing ridge tops in forests with a pine component and water sources nearby. We used a 30 m resolution for this modeling effort to best represent the dynamic and rugged topography of our study area and to make the best use of available landcover data from the National Park Service and the U.S. Forest Service. In the spatial model we created, forest type and elevation were the most important predictors of the distribution of suitable and optimal summer habitat for *M*. *sodalis* within our study area.

## Materials and Methods

### Study Area

**Fig 1:**
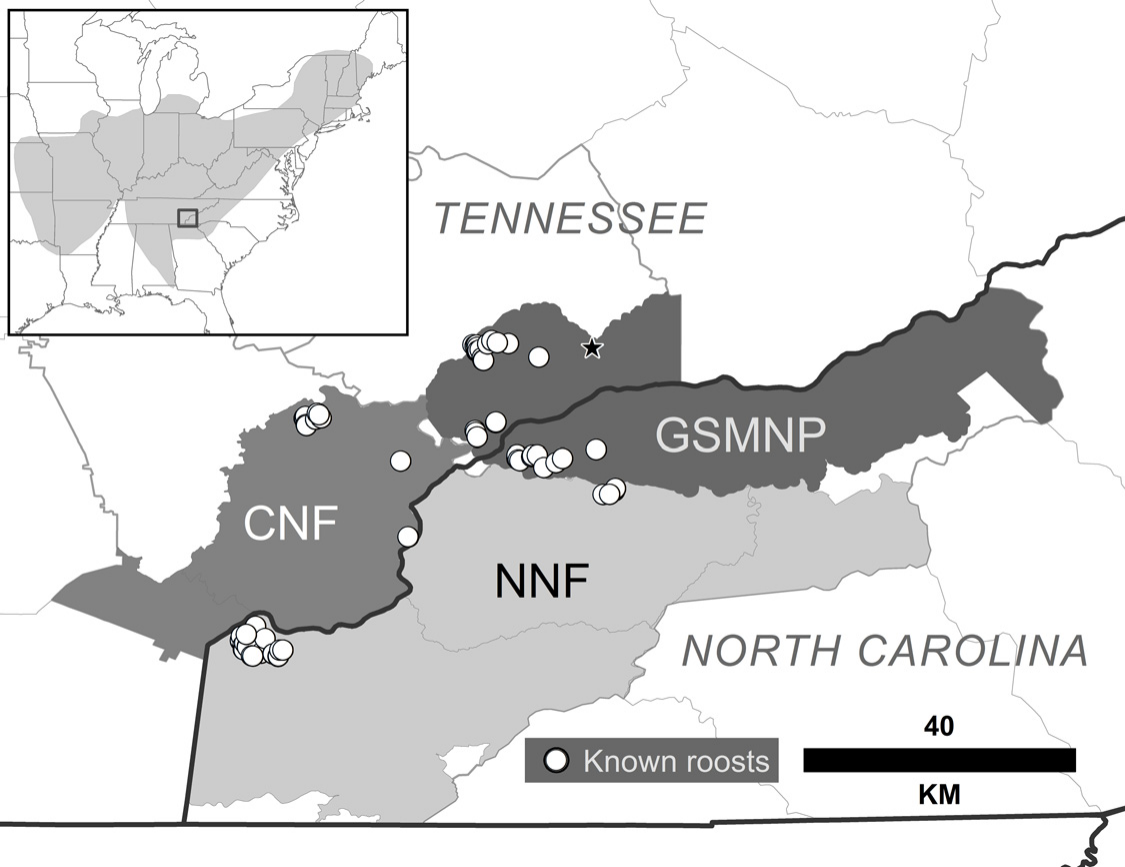
Locations of 76 roosts used by *Myotis sodalis*. Roosts for adult female and juvenile *Myotis sodalis* (May to August, 2008–2012) in the study area in the southern Appalachian Mountains (inset is *M*. *sodalis* distribution in eastern North America). A series of caves (star) are thought to be used as hibernation sites by *M*. *sodalis* that form summer colonies in this region. The study area (~281,800 hectares) included portions of the Great Smoky Mountains National Park (GSMNP), Nantahala National Forest (NNF), and Cherokee National Forest (NNF).

The study was conducted in the southern Appalachian Mountains in southeastern Tennessee and southwestern North Carolina in the Cherokee National Forest (CNF), the Nantahala National Forest (NNF), and Great Smoky Mountains National Park (GSMNP). The study area was 281,788 ha of federal land in Monroe and Blount counties, Tennessee, and Swain, Graham, and Cherokee counties, North Carolina (Fig 1). There were several known *M*. *sodalis* hibernacula in and around the GSMNP [10] and summer colonies of *M*. *sodalis* have been observed in the region since 1999 [9–10].

GSMNP and the two National Forests were classified as mixed pine *Pinus*-hardwood forests in the Appalachian oak forest region [30]. The primary natural community types used by bats were pine-oak *Quercus* heath, hemlock *Tsuga canadensis* forest, white pine *Pinus strobus* forest, low mountain pine-oak forest, and southern mountain xeric pine-oak woodland [12]. Various oak and cove forest types also occurred in our study area. The majority of the study area was forested habitat (> 90%), comprised mainly of mid-successional forest (41–80 years old), but also young and old-growth forests [31]. Elevation ranged from 260–2025 meters above sea level.

### Sampling

To locate day roosts for female and juvenile *M*. *sodalis*, we conducted mist netting surveys over trails, roads, and streams, and radio-telemetry from mid-May through mid-August 2008 to 2012. We banded bats with a unique 2.9 mm aluminum forearm band (Porzana Ltd., East Sussex, UK) for individual identification and fitted *M*. *sodalis* of suitable mass (>7 g for adult) with a 0.32–0.42 g radio transmitter (Holohil Systems Ltd., Ontario, Canada). We targeted adult females or juveniles due to their tendency to roost in colonies; up to 3 bats were transmittered on a given night. All bats were released at the capture site. We followed recommended white-nose syndrome protocols [32] and handling guidelines of the American Society of Mammalogists [33]. This work was approved by the Clemson University Institutional Animal Care and Use Committee (protocol 2009–16) and the Indiana State University Institutional Animal Care and Use Committee (protocol 226895–1). Field work was conducted under permits held by J O'Keefe: USFWS federal recovery permit TE206872, North Carolina permit ES261, Tennessee permit 3148, and National Park Service Permits GRSM-2009-SCI-0075 and GRSM-2012-SCI-0085.

We tracked bats using a TR5 receiver (Telonics, Mesa, AZ) and Yagi antennae (Wildlife Materials, Murphysboro, Illinois) the morning following capture and each day thereafter for the life of the transmitter (8–14 days), or until the bat was lost and unable to be relocated. We revisited all roost trees after mid-August each year to more extensively characterize and measure roost trees for comparison with model results and for another study on roost habitat selection. We recorded roost tree species or genus, tree height, roost height, diameter at breast height (dbh), and canopy closure directly above the roost to the nearest 25% interval. We recorded roost locations with a GEO-XT Trimble (Trimble Navigation Ltd., Sunnydale, California, USA) with sub-meter accuracy.

### Environmental data

MaxEnt requires known occurrence data to create distribution models. We corrected raw GPS roost locations using base station data in Pathfinder v.5.0 [Trimble Navigation Ltd., Sunnydale, California, USA], and imported points as occurrence data in MaxEnt (v3.3.3K, http://www.cs.princeton.edu/~schapire/MaxEnt/). To reduce the influence of closely clustered points, we identified a subset of 54 roosts for analyses by randomly selecting 1 roost from each of the 54 stands containing known roosts; stand boundaries were delineated in GIS shapefiles provided by the CNF, NNF, and GSM. Stands containing known roosts averaged 19.4 ± 3.2 ha in area. This method reduced spatial autocorrelation in our dataset and accounted for the fact that among stands there was often substantial variation in aspect, elevation, forest type, and other environmental factors as a result of the extremely rugged terrain in our study area.

We entered the following environmental layers into MaxEnt as predictors of the distribution of summer roosting habitat: elevation, aspect (compass direction), slope, distance-to-ridge, ridge curvature, distance-to-water, distance-to-major roads, distance-to-trails and closed roads, and forest type. We chose distance-to features that we thought may be important to bats as foraging resources, potential travel corridors, or for higher solar exposure (i.e., distance to ridgetop, [34]). We selected the topographic variables elevation, slope, aspect and curvature that are often used in landscape analyses for bat habitats (e.g., [8], [35]) and also to represent potential microclimate variation within the landscape.

All environmental layers were represented at a 30 m resolution in ArcGIS 10 [ESRI, Redlands, CA, USA] to maintain a high resolution base layer that would be usable by land managers with access to the same environmental layers. Forest type was the limiting layer in terms of area covered (only public lands) and resolution of data (30 m). Thus, we resampled all higher resolution GIS layers to 30 m to best represent this highly variable landscape. In early mapping tests, we found that resampling all the environmental layers to > 30 m resulted in a loss of variation among pixels in layers such as aspect and slope. We used the Euclidean Distance tool to generate all “distance-to” raster layers.

The categorical variable forest type was created from information provided by the CNF, NNF, and GSMNP. Due to differences in coding by agency, we generated unique codes for vegetation across the entire study area using primary and secondary vegetation species descriptions in Park Service and Forest Service layers and then merged the two based on the new code (S1 Table).

Elevation, aspect, slope, distance-to-ridge, and ridge curvature were generated from the digital elevation models (DEMs) for our study area (http://ned.usgs.gov, 1/3 arc-sec/10 m resolution). Due to its circular nature, aspect was separated into East/West and North/South by calculating the cosine and sine values. To measure slope, distance-to-ridge, and ridge curvature, we first removed production artifacts from DEMs through 10 successive smoothing filters run in Spatial Analyst (ESRI, Redlands, CA). We then generated slope and ridge-curvature with Spatial Analyst tools. Distance-to-ridge required further processing. Flow tools are typically used to identify low points in topography; however, we used the tools to identify high points and peaks (ridge tops) by multiplying outputs by -1. The base layer was filled to remove sinks, and then high and low values were reversed using Raster Calculator to multiply the field value by -1. We ran the Flow Direction tool, followed by the Flow Accumulation tool, and then generated a Stream Order raster to identify ridgelines.

Detailed water body (lakes and ponds) and stream water data were acquired from the National Hydrography Dataset (http://nhd.usgs.gov). We merged the flowline shapefiles (streams and rivers) and added a 1 m buffer to transform lines to a polygon feature to merge with the waterbody shapefile. The resulting vector file was then transformed into a 10 m raster. Major roads and trails/minor roads layers were developed from spatial data provided by the Park Service and Forest Service. Gated and closed roads were merged with trails, as these minor roads have minimal traffic/disturbance; major roads were any roads not included in the trails/minor roads layer. After clipping each environmental layer to the extent of the federal lands within our study area, we transformed the data into ascii files, the final data format required by MaxEnt.

We tested for collinearity amongst our environmental variables using the Band Collection Statistics Tool in ArcGIS. Due to the irregular shape of our study area (with null data in interior areas), it was not feasible to do this for the entire raster area. Instead, we clipped out two 1 km radius circles, one in the most rugged portion and one in the least rugged portion of our study area. Within each area, we assessed collinearity for the topographic variables in our analysis: aspect east, aspect north, curvature, distance to ridge, elevation, and slope. In the most rugged portion of the study area, distance to ridge and curvature were correlated. Curvature did not appear in any plausible models, but distance to ridge was in one top model. In the least rugged portion of the study area, distance to ridge was moderately correlated (< 0.7) with both aspect east and aspect north; these three variables appear together in the top ranked model in our analysis.

### Modeling

To avoid over parameterization of our model and, hence, model uncertainty [29], we used an information theoretic (IT) model selection procedure to select the most parsimonious model similar to the approach taken by Meyer et al. [36] and Pie et al. [37]. However, instead of using the permutation importance values to select variables to include in our models, we developed 26 candidate models, including a global model, for a set of potentially plausible, a priori hypotheses [29] (Table 1) based on existing information about roost habitat selection by *M*. *sodalis*. All variables were used eight times in total, for a balanced model set (Table 1) [38]; however, in keeping with the principle of parsimony imperative to the IT approach [38], and to reduce the risk of overfitting the data with too many parameters, 23 of the 26 candidate models contained only 1–4 variables. We also used the linear and quadratic features option in MaxEnt to avoid overfitting and to keep models comparable. We randomly selected 80% of the random subset of roosts (n = 43) to train each model, while 20% of roosts (n = 11) were used to test each model’s performance. To limit bias, test roosts were kept as a separate file so that the same sets of roosts were used to create and test all models. The lambda file (a text file of the parameter estimates) and ascii file from each model output and a text file of their pathways were input into ENMTools (v1.3) to generate an output of AIC scores corrected for small sample sizes (AIC_c_) [27]. Models with ΔAIC_c_ values < 7 were considered to have some support, but we also considered model weights relative to the entire candidate set when identifying top models [29]. For top models, we present AUC accuracy measures; however, we recognize the limitations of these measures and feel model performance is better assessed via an exploration of model reliability (agreement between predicted probabilities of occurrence and proportion of sites occupied [39]) and AIC_c_ model weights relative to the entire candidate model set. Parameter importance values were calculated across the entire candidate model set by summing the AIC_c_ model weights for each model in which a particular parameter appeared.

**Table 1:** Candidate models.

Model Name	Hypotheses	Variables^a^
Corridor 1	Closer to trails	TR
Corridor 2	On shallow ridges and closer to major roads	MR + C
Corridor 3	Closer to water	W
Elevation	At lower elevations	E
Foraging 1	Closer to water, trails, and major roads for foraging	W
Foraging 2	Lower slope, and closer to water and trails	S +W + TR
Forest Type	Uses forests with a pine-hardwood component	FT
Global	All variables are important to roost selection	FT + NS + EW + S + E + W + TR + MR + R + C
Humans	Near human made corridors	TR + MR
Major Roads	Near major roads	MR
Needs 1	At lower elevations near water	E + W
Needs 2	In pine-hardwood forests near water	FT + W
Needs 3	Near water, travel corridors, and pine-hardwood	FT + W + TR + MR
Pine 1	In conditions that promote pines	FT + E
Pine 2	In conditions that promote pines based on ridge location	FT + NS + EW + E + R
Research Bias 1	Near easy access trails/roads in forest types we targeted	FT + TR + MR
Research Bias 2	Near easy access trails/roads and ridgetop telemetry points	TR + MR + R
Ridge 1	South-facing, low slope, and near ridgetop	NS + EW + S + R
Ridge 2	Low slope, shallow ridges at lower elevation	S + E + C
Ridge 3	Near the ridgetop of shallow ridges in pine-hardwood forests	FT + R + C
Sun 1	Near ridgetop of shallow ridges with south-facing	NS + EW + R + C
Sun 2	On gentle slopes of shallow ridges with south-facing	NS + EW + S + C
Sunny Ridge top	Near the ridgetop and south-facing	NS + EW + R
Topography 1	Near the ridgetop on shallow, gentle slopes with south facing	NS + EW + S + E + R + C
Topography 2	Gentle south-facing slopes at lower elevations	NS + EW + S + E
Water flow	Shallow ridges at low elevations where water flows seasonally	S + W + C

Models developed to predict probability of presence of roost habitat used by *Myotis sodalis* in the southern Appalachian Mountains.

^a^Variables used in models were: forest type (FT), aspect north/south (NS), aspect east/west (EW), slope (S), Elevation (E), distance-to-water (W), distance-to-trails/minor roads (TR), distance-to-major roads (MR), distance-to-ridge (R), and ridge curvature (C).

We used raw map output from MaxEnt and the following logistic equation to create raster probability layers for plausible models:

$$logistic=(raw\;*e^{entropy})/(1+raw\;*e^{entropy})$$ (1)

We input raw values for each plausible model into Eq 1 in the Raster Calculator in ArcGIS to create logistic output maps and then created a weighted average map for the final raster output. Entropy, which is reported for each model, is the level of “choice” in a distribution; a higher entropy value means fewer constraints on distribution possibilities [15]. We chose a logistic output with suitability ranging from 0 (lowest) to 1 (highest) so that results would be easy to interpret and to facilitate comparisons with other habitat suitability models. When there is uncertainty over which model from the a priori set is the best model, it is good practice to base inferences on all of the plausible models in the set [29]. To increase the precision of our estimates [38], we created a final model raster layer using AIC_c_ model weights to average logistic outputs for top models with the Raster Calculator tool in ArcGIS. In the final model, areas where probability of presence was ≥ 0.5 were considered suitable habitat and areas where probability of presence was ≥ 0.75 were considered optimal habitat based on the definitions given by MaxEnt [40]. These conservative assumptions should minimize the commission error of our predictions, thus ensuring that the predicted areas all contain suitable roosting habitat for *M*. *sodalis*.

## Results

From 2008 to 2012 we captured and tracked 48 bats (45 adult female and 3 juvenile) *M*. *sodalis* to 76 roosts on federal land within our study area. We found 36 roosts in Tennessee (12 in Monroe County and 24 in Blount County), and 40 roosts in North Carolina (13 in Swain County, 4 in Graham County, and 23 in Cherokee County) (Fig 1).

Bats typically roosted under the sloughing bark of dead trees, most of which were ephemeral and only suitable for 1–2 years before losing all bark or falling to the ground. During the study, only one roost was used in two consecutive years; we noted fewer bats using this roost during exit counts in the second year. Roosts were primarily large diameter yellow pine *Pinus* subgenus *Diploxylon* (67% of roosts) or white pine snags (29% of roosts), of moderate height, and with low canopy closure (Table 2). We also located 2 hemlock roosts and 1 red maple *Acer rubrum* roost in this study. We observed that roosts were generally on south facing ridges, often on the upper third of the ridge (Table 2).

**Table 2:** Summary data for *Myotis sodalis* roosts.

Characteristic	Mean ± SE or Percentage	Minimum	Maximum
Tree Height (m)	19.9 ± 0.9	5.2	38.5
Dbh (cm)	39.8 ± 2.3	13.9	137.5
Canopy Closure %	25.5 ± 3.9	0	100
Elevation (m)	554 ± 21	266	1266
Aspect (degree°)	188° ± 9.1°	8°	344°
Slope Position	20% Lower, 45% Mid, 35% Upper	--	--
% Yellow Pine	68.4%	--	--
% White Pine	27.6%	--	--
% Hardwood	1.3%	--	--
% Hemlock	2.6%	--	--

Summary statistics for characteristics of 76 *Myotis sodalis* roosts located from May to August, 2008–2012 in the southern Appalachian Mountains.

Pine 2 (forest type + aspect north/south + aspect east/west + elevation + distance-to- ridge) ranked as the top model and Pine 1 (forest type + elevation) ranked second (ΔAIC_c_ = 3.3) when compared to 24 other candidate models (Table 3). Together Pine 2 and Pine 1 accounted for > 0.93 of the AICc weights and, therefore, there was a > 93% chance that one of them was the best approximating model for the occurrence data and candidate models we tested. AUC values produced from MaxEnt training and test data, Pine 2 (training AUC = 0.92, test AUC = 0.79) and Pine 1 (training AUC = 0.90, test AUC = 0.75) also suggested strong performance for both models.

**Table 3:** Model rankings.

Rank	Model	AIC_c_ Score	ΔAIC_c_	w_*i*_
1	Pine 2	1540.54	0	0.78
2	Pine 1	1543.81	3.27	0.15
3	Topography 1	1546.09	5.56	0.05
4	Topography 2	1548.07	7.53	0.02
5	Elevation	1559.21	18.67	< 0.01
6	Needs 1	1561.9	21.36	< 0.01
7	Global	1570.12	29.58	< 0.01
8	Research bias 1	1583.07	42.53	< 0.01
9	Needs 3	1586.23	45.69	< 0.01
10	Ridge 1	1593.89	53.35	< 0.01
11	Sun 2	1595.68	55.14	< 0.01
---	---	---	---	---
25	Corridor 1	1613.98	73.44	< 0.01
26	Corridor 3	1614.95	74.41	< 0.01

Eleven top-ranked models and the two lowest-ranked models for predicting the presence of *Myotis sodalis* summer roosting habitat in the southern Appalachian Mountains, May to August, 2008–2012. Data are for models testing a subset of 54 roosts located from May to August, 2008–2012 in the southern Appalachian Mountains. Models were ranked based on ΔAIC_c_.

**Fig 2:**
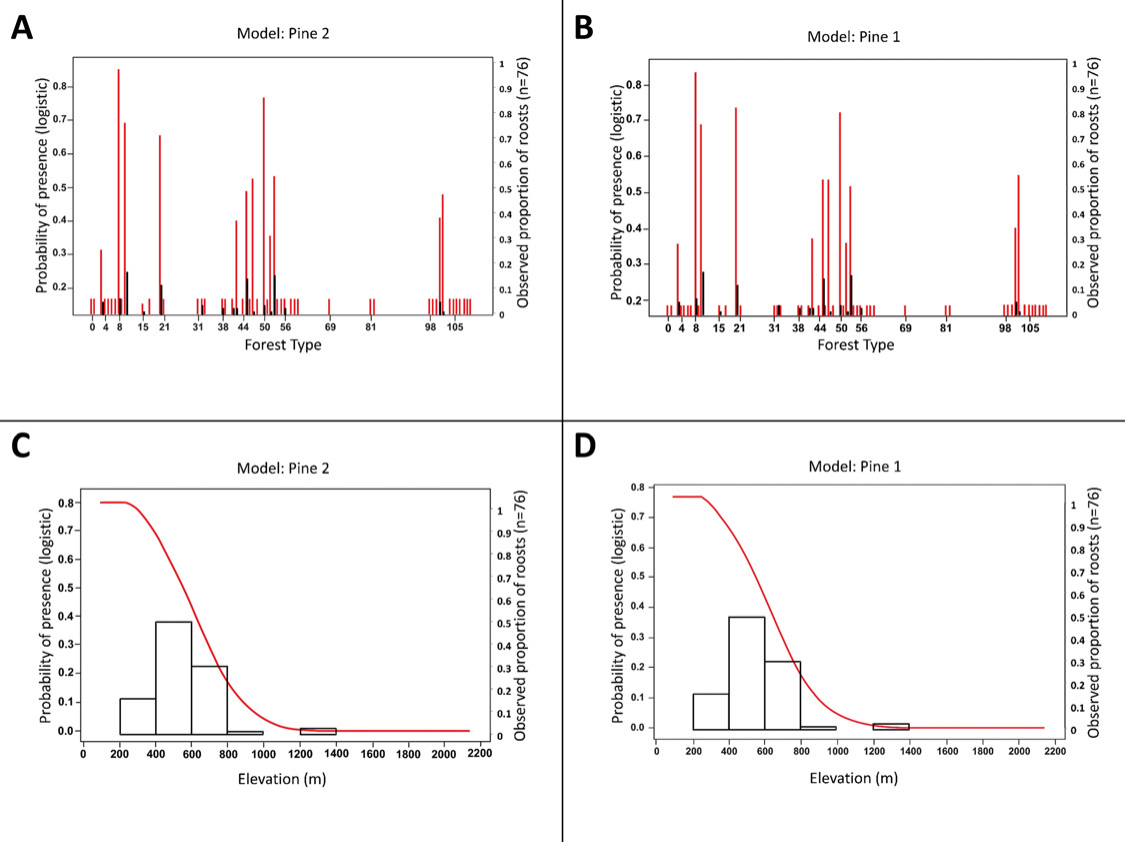
**Response curves (red) for forest type (A, B) and elevation (C, D) for top-performing models**. The observed proportions of roosts in each forest type or elevation class are plotted as black bars on secondary axes. The best models, Pine 2 and Pine 1, were developed using a subset of 54 female and juvenile *Myotis sodalis* roost locations from May to August, 2008–2012 in the southern Appalachian Mountains of North Carolina and Tennessee. See S1 Table for vegetation codes.

**Fig 3:**
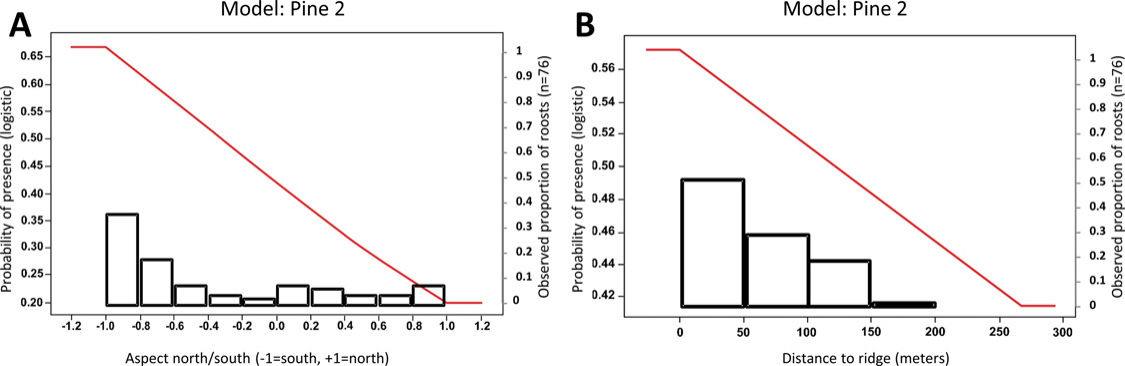
**Response curves (red) for aspect North/South (A) and distance-to-ridge (B) for Pine 2.** The observed proportions of roosts in each aspect or distance-to-ridge class are plotted as black bars on secondary axes. Pine 2 was one of the two best models developed using a subset of 54 roost locations for female and juvenile *Myotis sodalis* roosts from May to August, 2008–2012 in the southern Appalachian Mountains of North Carolina and Tennessee.

The five most important parameters (Table 4) were elevation (importance value = 1) and forest type (0.93), and aspect north/south (0.85), aspect east/west (0.85), and distance-to-ridge (0.83). For other variables, parameter importance values were < 0.067. Eight forest types (parameter estimate value ≥ 1.5; Table 5) were important in predicting the presence of summer roosting habitat, with 5 of these including a pine component: Hemlock-Hardwood, White Pine-Upland Hardwoods, Yellow Pine-Hardwoods, Oak-Yellow Pine, White Oak-Black Oak-Yellow Pine, Yellow Poplar, Oak-Hickory, and Yellow Pine (Fig 2A and 2B). The probability of presence of roosting habitat was > 0.5 for elevations ranging from 260 to 575 m (Fig 2C and 2D), though 29 of 76 known roost locations occurred at > 575 m in elevation; high elevation roosts included two hemlocks (1265 m elevation), one shortleaf pine *P*. *echinata* (815 m), one yellow pine unidentifiable to species (795 m), and one Table Mountain pine *P*. *pungens* (765 m). Probability of presence was >0.5 for south-facing slopes, decreasing as aspect became more north-facing (Fig 3A). The response curve for aspect east/west did not show strong directionality for probability of presence; it was important merely because it was used in the same models as aspect north/south. Lastly, the response curve for distance-to-ridge showed that probability of presence of roosting habitat was > 0.5 at points <125 m from the top of the ridge, with decreasing probability of presence as distance-to-ridge increased (Fig 3B).

**Table 4:** Variable importance values.

Environmental Variables	Parameter Importance
Elevation	1
Forest Type	0.93
Aspect East/West	0.85
Aspect North/South	0.85
Distance to Ridge	0.83
Slope	0.07
Curvature	0.05
Distance-to-Major Rds	<0.001
Distance-to-Trails/Minor Rds	<0.001
Distance-to-Water	<0.001

Importance values for variables used in 26 candidate models predicting the presence of *Myotis sodalis* summer roosting habitat in the southern Appalachian Mountains, May to August, 2008–2012. Importance values for each variable were based on the AICc weights for each model in which a variable was included.

**Table 5:** Parameter estimates for two top-ranked models.

Environmental Variables	Pine 2 Parameter Estimate 1	Pine 1 Parameter Estimate 2
White Pine	0.81	0.88
**Hemlock-Hardwood**	**3.31**	**3.1**
**White Pine-Upland Hardwood**	**2.39**	**2.28**
Yellow Pine-Oak	-0.12	---
**Yellow Pine-Hardwoods**	**2.23**	**2.50**
Upland Hardwoods-White Pine	1.17	0.95
**Oak-Yellow Pine**	**1.54**	**1.63**
**White & Black Oak, & Yellow Pine**	**1.68**	**1.62**
**Yellow Poplar**	**2.77**	**2.44**
Chestnut Oak	1.00	0.91
**Oak-Hickory**	**1.72**	**1.55**
Early Successional Hardwoods	1.22	1.08
**Yellow Pine**	**1.50**	**1.67**
**Elevation**	**-3.24**	**-2.02**
**Elevation^**^**2**^	**-13.04**	**-13.93**
**Aspect North/South**	**- 2.10**	---
Distance to Ridge	-0.63	---
Distance to Ridge^^2^	---	---
Aspect East/West	0.35	---
Aspect East/West^^2^	0.22	---
**MaxEnt Parameters**		
Linear Predictor Normalizer^a^	2.22	2.86
Density Normalizer^b^	235.64	274.42
Entropy^c^	7.90	8.12

Parameter estimates for each environmental variable in the two top-ranked models predicting the probability of presence of *Myotis sodalis* roosting habitat in the southern Appalachian Mountains, May to August, 2008–2012. These parameter estimates, along with the normalizers and entropy values, were used to create the final raw and logistic equations for each MaxEnt model. Forest types with parameter estimates >1.5 and other important environmental variables are bolded.

^a^Constant chosen so that the exponent is always non-positive (for numerical stability).

^b^Constant that ensures that all possibilities of distribution sum to one.

^c^Level of “choice” in a distribution.

**Fig 4:**
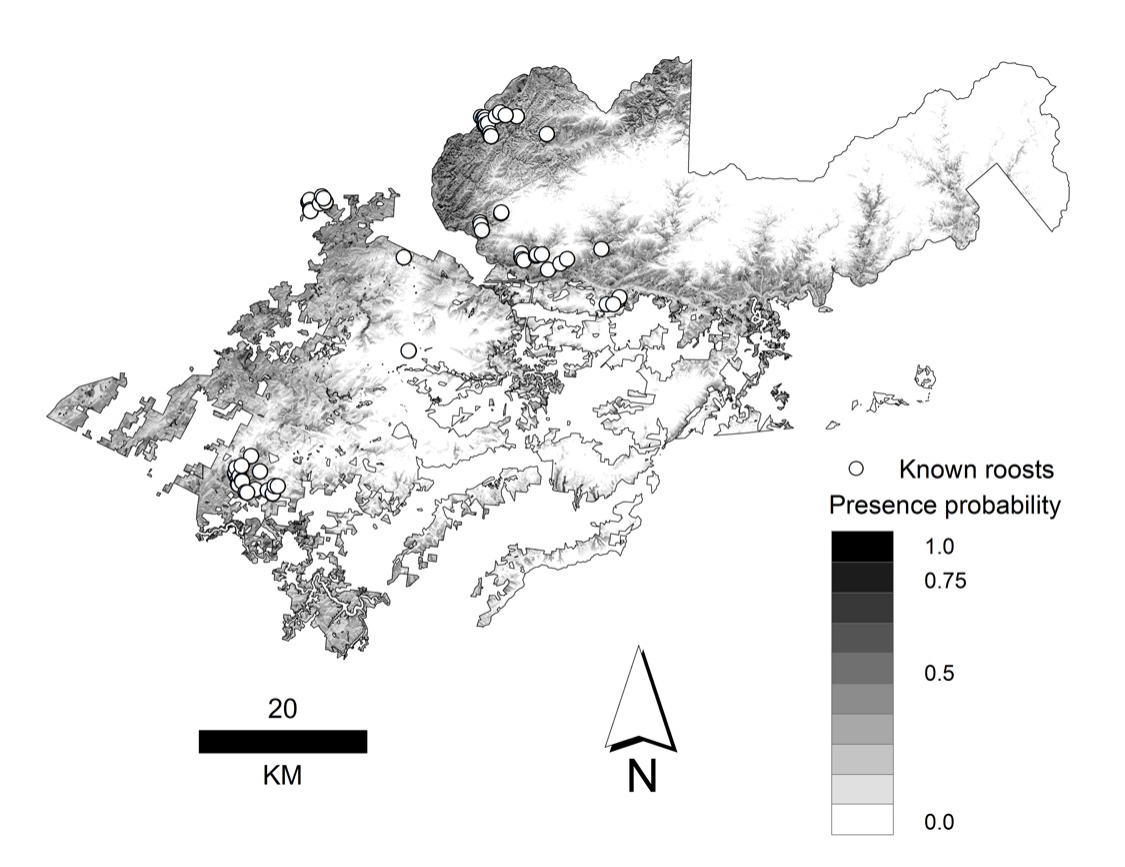
Predicted probability of the presence of summer roosting habitat for *Myotis sodalis*. Probability map is based on the average logistic model and shows 76 roosts used by female and juvenile *Myotis sodalis* from May to August, 2008–2012 in the southern Appalachian Mountains of North Carolina and Tennessee. Areas of importance (dark gray to black areas) are either suitable (≥ 0.5) or optimal (≥ 0.75) summer roosting habitat.

The final probability raster (Fig 4) shows predicted areas of suitable (5% of study area) and optimal (0.5% of study area) habitat for the period 2008–2012. Suitable and optimal cells were located in areas with known roosts, but also in areas where no roosts were located (Fig 4). From the original set of 76 known roosts, 24 roosts were located within areas predicted to be suitable habitat and 9 roosts were within areas predicted to be optimal habitat.

## Discussion

The spatial model was successful in predicting the distribution of habitat important to *M*. *sodalis* during the time of our study, and made predictions beyond surveyed areas. The final average model identified forests at elevations 260–575 m on the upper portions of south-facing slopes, mainly with a pine component, as important *M*. *sodalis* summer roosting habitat in the mountains of southeast Tennessee and southwest North Carolina. This contradicts findings in other portions of the species’ range where *M*. *sodalis* primarily uses bottomland hardwood forests or riparian areas (e.g., [5], [41–42]). Distance to water was not a good predictor of suitable habitat in this study, whereas hydric forests are preferred in the Midwest [5]. Differences between this study and others with respect to landscape-level roost selection suggest that *M*. *sodalis* is flexible in roost habitat selection across different ecoregions with varying topography and land use patterns [1], [42]. The model we produced should be useful within the southern Appalachians, but will not easily transfer to other ecoregions within the range of *M*. *sodalis*.

Bats often show high fidelity to and switch roosts within areas of suitable habitat, suggesting that landscape variables may be important in determining the suitability of an area (e.g., [1], [43–45]). Few studies have investigated roost habitat distribution on a landscape level [1]; most have focused on land cover data, separating these data into general categories (e.g., bottomland vs. coniferous forest, [41]; pine vs. hardwood and edge vs. open, [45]; and forest vs. wooded pasture, [42]). In this study, we found that topographical features and detailed data on forest composition were important predictors of the probability of presence of suitable roost habitat when considered over a large area (>200,000 ha).

The top models predicted summer roosting habitat to be primarily in forest types with a pine component, which supports previous work on plot- and stand-scale roost selection by *M*. *sodalis* in this same region [9]. Yellow pine species in this region tend to occur with oaks on ridgetops and south-facing (SW, S, SE) slopes where soil conditions are driest [12], [46], thereby providing good solar exposure, which is important for tree-roosting bats [4]. Oaks were not used in this study, but 96% of known roosts were in yellow or white pine snags. Pine species used by bats in our study area exhibited similar patterns of decay, with exfoliating bark remaining attached to the bole of the tree for several months, which is an important characteristic for Indiana bat roosts [42].

Elevation influences bat species distributions [47] and roost locations (e.g., [48]). Cryan et al. [13] suggest variation in temperature and insect availability at different elevation gradients may impact torpor and energy restrictions. Elevation influences the temperature regimes that a bat experiences while roosting and, thus, the amount of energy a bat expends for thermoregulation. The association with elevation may also be due to the presence of pine forests within a particular elevation band. Probability of presence of summer roosting habitat was greatest for elevations between 260–575 m, which includes the elevation gradient ranges for all of the pine species within the southern Appalachians [49]. Throughout the species’ range, *M*. *sodalis* maternity roosts are most likely to occur between 120 m and 330 m [22]; however, bats may favor higher elevations for cooler summer temperatures in the southern portion of the range [8], [this study].

While elevation and forest type were important for determining the general locations of potential suitable roosting habitat, including aspect and distance-to-ridge in the model further narrowed the range of areas predicted to provide optimal conditions for pine growth and greater solar exposure/optimal microclimates for reproductive females and their pups. The topography in the southern Appalachian Mountains can change drastically over short distances, with sunny ridges adjacent to deep drainages that are visibly cooler and shadier; thus, topography affects temperature regimes for roost habitat. Furthermore, south-facing aspects and the upper portion of ridges provide favorable growth conditions for several pine species [46]. The significance of topographic features in the southern Appalachian Mountains is highly contextual; this model will not transfer easily to areas with little topographical relief, such as the Midwestern U.S.

Some spatial and temporal factors were not included in the models we developed, but could be important for future modeling efforts. The inclusion of snag distribution and forest canopy closure data, which can be derived from high-resolution LiDAR (Light Detection and Ranging) data (e.g. [50]), might narrow the spatial scale of predictions. The development of standardized, high-resolution forest classification data would improve our ability to develop models that span large spatial scales and properties under management by different entities. Climate change may be a particularly important temporal factor, as tree species’ ranges and abundance may shift [51] and *M*. *sodalis* may shift the core of their range [22].

Spatial autocorrelation in occurrence points results in disparities in test and training performance measures for habitat distribution models and may lead to overfitting model parameters [35], [52]. Reducing spatial autocorrelation amongst occurrence points is essential when modeling species’ distributions across large areas where there are potential gaps in species’ occurrence. We did not fully eliminate the spatial clustering in our known occurrence data because we were hesitant to make predictive models using very few data points in highly variable terrain and because we felt that fully eliminating clustering ignored the biology of *M*. *sodalis*. In a preliminary analysis, we determined that randomly eliminating all points that were spatially autocorrelated (e.g., [20], [24]) would have left us with only 10 presence points > 5 km apart (where clustering began to decline for known roosts in this study) and given more weight to outlier points (e.g., 1 of 2 high elevation hemlock snags). Due to the ruggedness of our landscape, a short distance (< 500 m) between roosts typically coincided with a large change in topographic variables such as slope, aspect, and elevation. It is likely that modeling habitat distribution with a small sample of points spaced > 5 km apart would yield unstable predictions because model predictions may be unstable when a small sample does not fairly represent the environmental variation in the study area [17]. Furthermore, groups of *M*. *sodalis* in a maternity colony tend to switch amongst roosts in relatively close proximity and to select for particular habitat conditions within the landscapes they occupy (e.g., wetlands in an agricultural-forest matrix in Michigan) [7], which suggests it may be important to consider areas where potential roosts are clustered (patches of dead pines in this study) when modeling potential roosting habitat. Another way to reduce effects of spatial autocorrelation is to use a larger cell size for models (e.g., [20]). Due to the extremely rugged and variable nature of the terrain in our study area, using a larger grid cell size for this analysis would render most of our environmental variables meaningless (e.g., aspect, elevation, slope, and distance to ridge).

Despite the fact that we used somewhat clustered presence points, we believe that our models were not highly biased for overfitting for several reasons. First, restricting MaxEnt to use only linear and quadratic features minimized the likelihood of overfitting because our models were not overly complex [52]. Second, using the information theoretic approach with a priori hypotheses gives more parsimonious models (i.e., with fewer parameters) an advantage over more complex models and reduces concerns about overfitting [29]. Finally, only 43% of known roosts were in areas predicted to be optimal or suitable and the final model predicted occupancy in many areas where there were no roosts (Fig 4). Indeed, in 2015, we tracked a male *M*. *sodalis* to 2 roosts located in an area predicted to be optimal habitat by our model that is > 40 km east of the closest roost used to develop the models in this study [O’Keefe et al., unpublished data].

Though this model is static, bat roost choice and habitat selection are dynamic. Thus, specific areas of suitable and optimal habitat will change over time. Known pine snag roosts are thought to have died as a result of a southern pine beetle *Dendroctonus frontalis* outbreak in the early 2000s [53], but mature yellow pine forests are becoming increasingly rare on the landscape [46]. In the absence of preferred roost types, *M*. *sodalis* will use other tree species as roosts [7], [9]. *T*. *canadensis* snags are currently readily available due to widespread die-offs from the invasive wooly adelgid, *Adelges tsugae* [54]; however, to date, we have observed low use of these trees. Management practices that promote forests with a pine component on upper south-facing slopes at elevations from 260–575 m and create or preserve large trees should yield suitable roosting habitat for *M*. *sodalis*. Prescribed fire could be an important tool for restoring *M*. *sodalis* habitat, but it will be important for managers to consider the potential for regrowth of pine species in burned areas and how to promote large trees with good roosting conditions in the future.

Mist netting and radio tracking are not always economically and logistically feasible means to locate bat roosts [55]. Model creation and comparison based on known occurrence data provides a supplemental, if not alternative, way for managers to identify important *M*. *sodalis* summer roosting habitat areas and factors important to roost habitat selection. This approach is proactive in that it will facilitate management for future habitat through the identification of important environmental conditions. Landscape-scale GIS models predicting the distribution of suitable habitat may also be useful for other forest bat species [1].

## Supporting Information

S1 Table Vegetation codes for landcover types on USFS and NPS lands. Vegetation codes for landcover types on USDA Forest Service (USFS) and National Park Service (NPS) lands. Codes were inputs in models developed using 54 of 76 known roost locations for female and juvenile *Myotis sodalis* roosts from 2008–2012 in the southern Appalachian Mountains of North Carolina and Tennessee. We combined codes because the NPS often grouped forest types, while the USFS codes were more species-oriented. Blank cells indicate distinct landcover types found only on either USFS or NPS land, not both.
(DOCX)
