# Supplementary material for: A Presence-Only Model of Suitable Roosting Habitat for the Endangered Indiana Bat in the Southern Appalachians
Source: PLoS One. 2016 Apr 26;11(4):e0154464. doi: 10.1371/journal.pone.0154464 (PMC4846014; doi:10.1371/journal.pone.0154464)
Supplement: S1 Table — Vegetation codes for landcover types on USDA Forest Service (USFS) and National Park Service (NPS) lands. Codes were inputs in models developed using 54 of 76 known roost locations for female and juvenile Myotis sodalis roosts from 2008–2012 in the southern Appalachian Mountains of North Carolina and Tennessee. We combined codes because the NPS often grouped forest types, while the USFS codes were more species-oriented. Blank cells indicate distinct landcover types found only on either USFS or NPS land, not both. (DOCX) [file pone.0154464.s001.docx]

**S1 Table. Vegetation codes for landcover types on USFS and NPS lands**

Vegetation codes for landcover types on USDA Forest Service (USFS) and National Park Service (NPS) lands. Codes were inputs in models developed using 76 known roost locations for female and juvenile Indiana bat roosts from 2008-2012 in the southern Appalachian Mountains of North Carolina and Tennessee. We combined codes because the NPS often grouped forest types, while the USFS codes were more species-oriented. Blank cells indicate distinct landcover types found only on either USFS or NPS land, not both.

| Code For Models | USFS Landcover Types | NPS Landcover Types |
| --- | --- | --- |
| 0 | Undefined, or Water |  |
| 1 | Human Influence- road, building etc | Human influence, mud/gravel, road |
| 3 | White Pine | Eastern White Pine |
| 4 | White Pine-Hemlock | Eastern Hemlock-Eastern White Pine -Rhododendron |
| 5 | Hemlock | Hemlock Forest (typic type) |
| 6 | Fraser Fir | Fraser Fir |
| 7 | Red Spruce-Fraser Fir | Red Spruce-Fraser Fir |
| 8 | Hemlock-Hardwood | Red Maple-Sweet,Yellow Birch-Fraser Magnolia-Blackgum-Sourwood / Rhododendron |
| 9 | White Pine-Cove Hardwood |  |
| 10 | White Pine-Upland Hardwood | Eastern White Pine–Mesic Oak Forest & White Pine-Chestnut Oak-Scarlet Oak |
| 15 | Yellow Pine-Oak | Southern yellow pine species & White Oak-Red Oak-Chestnut Oak-Hickory or Chestnut Oak or Submesic to Mesic Oak/Hardwoods |
| 17 | Red Spruce-Northern Hardwoods |  |
| 20 | Yellow Pine-Hardwoods | Southern Yellow Pine species & Chestnut Oak-Hardwoods |
| 21 | Longleaf Pine |  |
| 31 | Loblolly Pine |  |
| 32 | Shortleaf Pine | Shortleaf Pine |
| 33 | Virginia Pine | Virginia Pine |
| 38 | Pitch Pine | Pitch Pine |
| 39 | Table Mountain Pine | Table Mountain Pine |

Table S1 Continued. Vegetation codes for landcover types on USFS and NPS lands.

| Code for models | USFS Landcover Types | NPS Landcover Types |
| --- | --- | --- |
| 41 | Cove Hardwoods-White Pine-Hemlock | S. Appalachian Cove Hardwood mixed with *Liriodendron*, Hemlock, White Pine, Yellow Birch-Northern Hardwoods, Oak-Hardwoods with Pines |
| 42 | Upland Hardwoods-White Pine | Early Successional Hardwoods or Oak/Hardwoods & White Pine |
| 44 | Southern Red Oak-Yellow Pine |  |
| 45 | Chestnut Oak-Scarlet Oak-Northern Red Oak-Yellow Pine | Oak-Hardwoods-Yellow Pine & Chestnut Oak-Hardwoods-Yellow Pine |
| 46 | Bottomland Hardwood-Yellow Pine |  |
| 47 | White Oak-Black Oak-Yellow Pine |  |
| 48 | Northern Red Oak-Hickory-Yellow Pine |  |
| 50 | Yellow Poplar |  |
| 51 | Post Oak-Black Oak |  |
| 52 | Chestnut Oak | Chestnut Oak & Chestnut Oak-Hardwoods |
| 53 | White Oak-Northern Red Oak Hickory | White Oak-Red Oak-Chestnut Oak-Hickory |
| 54 | White Oak | White Oak |
| 55 | Northern Red Oak |  |
| 56 | Yellow Poplar-White Oak-Northern Red Oak |  |
| 57 | Scrub Oak |  |
| 58 | Sweet Gum-Yellow Poplar | American Hornbeam Thicket & Sweetgum |
| 59 | Scarlet Oak |  |
| 60 | Chestnut Oak-Scarlet Oak | Chestnut Oak-Hardwoods & Red Oak-Red Maple-Mixed Hardwoods, Northern Red Oak & Chestnut Oak |
| 62 | Sweet Gum-Nuttail Oak-Willow |  |
| 69 | Beech-Magnolia | Beech Gap & Magnolia |
| 81 | Maple-Beech-Yellow Birch | Tuliptree-Red Maple-Sweet Birch & Cove Hardwoods-Yellow Birch |
| 82 | Black Walnut |  |

Table S1 Continued. Vegetation codes for landcover types on USFS and NPS lands.

| Code for models | USFS Landcover Types | NPS Landcover Types |
| --- | --- | --- |
| 88 | Black Locust |  |
| 98 | Undrained Flatwoods/Alluvial- includes so-called savannahs. Usually non-stock with management species. Stocking efforts complicated by water table at or nearly at surface throughout most of year. | Montane Alluvial Forest & Alluvial Habitats with non-forested sections |
| 99 | Brush and Shrub Species- Areas stocked with brush other than Bear Oak or the Southern Scrub Oaks; includes Rhododendrom and Mountain Laurel. | S. Appalachian Heath Balds, Rhododendron, Blackberry thickets, Cultivated Meadow, Old Field, Graminoid, Herbaceous forbs, and/or Mountain Laurel |
| 100 | Cove Hardwoods | S. Appalachian Cove Hardwoods & Northern Red Oak Cove Forest |
| 101 | Early Successional Hardwoods | S. Appalachian Early Successional Hardwoods |
| 102 | Yellow Pine | Yellow Pine |
| 103 | Exotic | Exotic Vegetation |
| 104 | Graminoid/Old Meadows | Old Field & Graminoids |
| 105 | Grassy Bald | High Elevation Grassy Bald, Mountain Oatgrass-Mountain Cinquefoil-Herbaceous & Rock Outcrops/Summits |
| 106 | Successional Vegetation | Disturbed Northern Hardwood Woodland & Successional Vegetation |
| 107 | Northern Hardwoods | Northern Hardwoods |
| 108 | Succesional Hardwoods | Successional Hardwoods |
| 109 | Yellow Pine/White Pine mix | Yellow Pine species & White Pine mix |
| 110 | Spruce Hemlock mix | Spruce-Hemlock |
